# HDAC inhibitors stimulate viral transcription by multiple mechanisms

**DOI:** 10.1186/1743-422X-5-43

**Published:** 2008-03-19

**Authors:** Lata Balakrishnan, Barry Milavetz

**Affiliations:** 1 Department of Biochemistry and Molecular Biology, University of North Dakota, Grand Forks, North Dakota, USA

## Abstract

**Background:**

The effects of histone deacetylase inhibitor (HDACi) treatment on SV40 transcription and replication were determined by monitoring the levels of early and late expression, the extent of replication, and the percentage of SV40 minichromosomes capable of transcription and replication following treatment with sodium butyrate (NaBu) and trichostatin A (TSA).

**Results:**

The HDACi treatment was found to maximally stimulate early transcription at early times and late transcription at late times through increased numbers of minichromosomes which carry RNA polymerase II (RNAPII) transcription complexes and increased occupancy of the transcribing minichromosomes by RNAPII. HDACi treatment also partially relieved the normal down-regulation of early transcription by T-antigen seen later in infection. The increased recruitment of transcribing minichromosomes at late times was correlated to a corresponding reduction in SV40 replication and the percentage of minichromosomes capable of replication.

**Conclusion:**

These results suggest that histone deacetylation plays a critical role in the regulation of many aspects of an SV40 lytic infection.

## 1. Background

Structural and biological changes in chromatin structure are brought about by changes in the activity of histone acetyltransferases (HATs) and histone deacetylases (HDACs) which add or remove acetyl groups from lysine residues on histone tails respectively. It has been well established that inhibition of HDAC activity is characterized by two important changes within the cell (i) an increase in the amount of hyperacetylated histones [1] and (ii) an increase in the level of transcription of certain genes [2,3]. However we know little about the specific mechanisms underlying the relationship between HDAC inhibition (HDACi) and alterations in gene expression at the molecular level. Since remodeling of chromatin structure plays a vital role in the regulation of gene expression [4] the enzymes involved in this modification process have been used as common targets to alter the pattern of gene expression.

Sodium butyrate (NaBu) and Trichostatin A (TSA) are commonly used reversible inhibitors of HDAC activity. NaBu, a short chain fatty acid occurring naturally in the body, is a byproduct of anaerobic bacterial fermentation of dietary fiber [5,6]. TSA, a hydroxamic acid is a fermentation product of *Streptomyces *and a potent inhibitor of HDAC activity. NaBu has been extensively used as a HDAC inhibitor (HDACi), though it is far less efficient (required in millimolar quantities) in its inhibition capabilities as compared to TSA (required only in nanomolar quantities). DNA micro array studies have shown that almost 7 % to 10% of genes undergo altered gene expression on HDACi using TSA [7-9]. HDAC inhibitors are now at the forefront of clinical trials for treatment of various types of tumors [10,11] either alone or in combination with other therapies.

During the course of our recent studies investigating the relationship between histone hyperacetylation and transcription in the SV40 minichromosome model system [12] we utilized NaBu as an HDACi in part to determine whether histone hyperacetylation was dynamic in a coding region undergoing active transcription [12]. In these studies we observed that there was a significant increase in the extent of histone hyperacetylation in a coding region undergoing active transcription following treatment with NaBu. However, we did not determine whether the increase in histone hyperacetylation was associated with an increase in transcription as might have been expected from the literature or how such an increase in transcription might occur in the SV40 model system.

We hypothesized that if SV40 transcription increased there were two simple ways that inhibition of histone deacetylation could lead to an increase in transcription. Either there could be an increase in the number of SV40 minichromosomes which carry RNAP II and undergo transcription, or the number of transcribing minichromosomes remained constant but there was an increase in the density of RNAPII on those minichromosomes with a corresponding increase in the extent of transcription.

We have now determined the effects of HDACi treatment with NaBu and TSA on SV40 early and late transcription and replication. Since both transcription and replication appeared to be affected, we have also determined the effects of HDACi treatment on the percentage of SV40 minichromosomes undergoing transcription and replication and the density of RNAPII on transcribing minichromosomes. These studies indicate that inhibition of HDAC activity can alter an SV40 infection by targeting a number of different functions of SV40 minichromosomes.

## 2. Results

### 2.1. Stimulation of SV40 transcription following inhibition of histone deacetylation

We have previously shown that treatment of SV40 infected cells with the HDACi NaBu resulted in a large increase in the amount of hyperacetylated H4 and H3 associated with transcribing SV40 minichromosomes [12]. Since treatment with an HDACi frequently results in the stimulation of transcription of receptive genes [13], we first determined whether NaBu treatment had a similar effect on SV40 early and late transcription. In order to exclude the possibility that the effects observed with NaBu were specific to this inhibitor and not a result of the inhibition of histone deacetylation, we also used a structurally distinct second HDACi, trichostatin A [14], in our studies.

In order to determine the effects of HDACi treatment on SV40 transcription, total proteins and mRNA were isolated 30 minutes post-infection (early) and 48 hours post-infection (late) from control and HDACi treated SV40 infected cells and analyzed by Western blotting and real time RTPCR. The proteins were analyzed with antibodies to T-antigen, to measure the effect on early transcription, VP-1, to measure the effect on late transcription, and glyceraldehyde-3-phosphate dehydrogenase (GAPDH) as a loading control. Similarly, mRNA was quantitated using primer sets that recognize the VP-1, T-antigen, and GAPDH mRNAs respectively.

During preliminary studies with various concentrations of HDACi we observed that treatment with 250 μM NaBu and 120 nM TSA resulted in significant increases in the expression of both T antigen and VP1 at appropriate times, without altering the expression pattern of GAPDH (data not shown).

**Figure 1:**
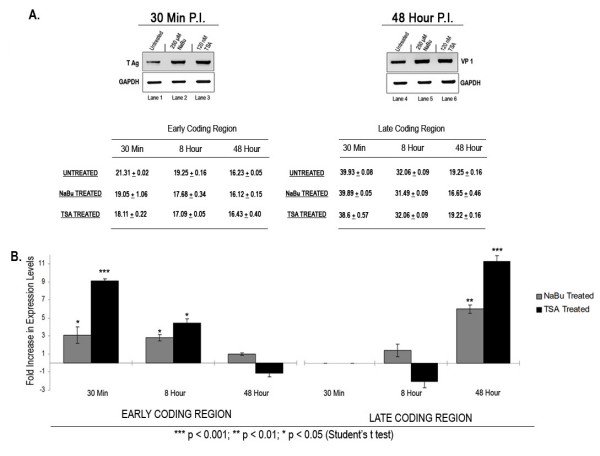
**Effects of HDACi treatment on SV40 transcription**. **(a) **Total protein extracted from 30 min and 48 hour SV40 infected BSC-1 cells and either untreated or treated with 250 μM NaBu or 120 nM TSA were separated using SDS PAGE followed by western blot analysis using antibody against T antigen (for 30 mins), VP1 (for 48 hours) and GAPDH (for both 30 mins and 48 hours). **(b) **Total RNA extracted from 30 min, 8 hour and 48 hour SV40 infected BSC-1 cells untreated and treated with 250 μM NaBu or 120 nM TSA were reverse transcribed and the amount of mRNA for T antigen and VP1 were analyzed using real time PCR using primer sets against early the coding region and late coding region, respectively. Data is expressed as the fold increase in expression levels compared to the mRNA from untreated SV40 infected BSC-1 cells and represent the average of three independent experiments.

Typical examples of the results of a Western blotting analysis are shown in Figure 1A. Following treatment of SV40 infected cells early in infection (30 mins post infection) with NaBu (lane 2) and TSA (lane 3) we observed an increase in the amount of T-antigen present in the cells compared to an untreated control (lane 1). Similarly, treatment with NaBu (lane 5) and TSA (lane 6) late in infection (48 hours post infection) resulted in an increase in the amount of VP1 compared to the untreated control (lane 4). We did not observe any significant changes in the amount of GAPDH present in the cells at early times (compare lanes 1–3) or late times (compare lanes 4–6) following treatment with either HDACi.

In order to confirm that the changes in the amount of late and early proteins in the Western blot analysis following HDACi treatment were a result of an increase in the amount of mRNA and not an effect on protein stability, we next determined the levels of mRNA for VP-1 and T-antigen in treated and control SV40 infected cells. Because T-antigen undergoes down-regulation at approximately 8 hours post-infection we also analyzed mRNA expression at this time in order to determine whether the inhibitors affected early transcription during down-regulation.

Total RNA was prepared from treated and untreated SV40 infected cells and subjected to real-time RTPCR analysis with (Figure 1B). Based upon the change in cycle threshold from the real-time RTPCR analysis, we then calculated the fold increase in transcription at each of the time points for the treated cells compared to the controls. In untreated SV40 infected cells as expected, we found early message present throughout the infection and late message absent at 30 minutes post-infection, present in extremely small amounts at 8 hours post-infection, and present in large amounts at 48 hours post-infection (C_T _values for treated and untreated samples are indicated in the figure). The data shown is the average of at least three separate analyses of treated and untreated samples prepared in parallel.

At 30 minutes post-infection we observed a 3 fold increase in early message following treatment with NaBu and a 9 fold increase following treatment with TSA. We observed no late transcription at this time and no subsequent effect following treatment with the HDACi's. At 8 hours post-infection treatment with NaBu and TSA resulted in a 2.5 fold and a 4.5 fold increase in early message, respectively. At this time we again observed little late transcription and little effect on late transcription with either HDACi. In contrast, at 48 hours post-infection we observed little effect on the amount of early message present but a 6 fold increase with NaBu treatment and an 11 fold increase with TSA treatment on the amount of late mRNA. We did not observe any significant effects on the amount of GAPDH mRNA at any of the times following HDACi treatment (data not shown). Taken together, the Western blotting and real-time RTPCR analyses indicated that the HDACi treatment stimulated early and late SV40 transcription like many other genes, but that the stimulation occurred maximally when the genes were being actively transcribed.

### 2.2. Inhibition of histone deacetylation results in an increase in the number of transcribing SV40 minichromosomes

Since the increase in SV40 early and late mRNA and protein following HDACi treatment could be due to an increase in the number of SV40 minichromosomes undergoing transcription, we first determined whether HDACi treatment affected the number of SV40 minichromosomes carrying RNAPII, an enzyme absolutely required for eukaryotic transcription. We have previously shown that SV40 minichromosomes containing RNAPII can be immune selected with antibody to RNAPII in a ChIP assay for subsequent studies [12,15] and that the RNAPII on the SV40 minichromosomes is organized in a pattern consistent with its role in transcription [15].

**Figure 2:**
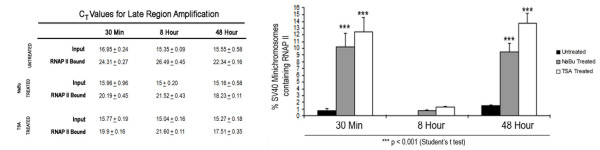
**Effects of HDACi treatment on chromosomes carrying RNA Polymerase II**. 30 min, 8 hour and 48 hour SV40 minichromosomes either untreated or treated with 250 μM NaBu or 120 nM TSA were immune selected using 10 μl of antibody against RNAPII. Data is expressed as the percentage of SV40 minichromosomes containing RNA Polymerase II and represent the average of three independent experiments.

SV40 minichromosomes from control and HDACi treated infected cells were harvested in parallel at 30 minutes, 8 hours, and 48 hours post-infection from at least three separate preparations of SV40 minichromosomes at each time point and subjected to a ChIP analysis with antibody to RNAPII followed by real-time PCR amplification for quantitation. From the C_T _values obtained from the analyses of HDACi treated and untreated SV40 minichromosomes we then determined the percentage of the SV40 minichromosomes that contained bound RNAPII from each set of samples (Figure 2).

In untreated SV40 minichromosomes we observed approximately 0.84 ± 0.19% of the total pool of SV40 minichromosomes carrying RNAPII at 30 minutes, 0.04 ± 0.01% at 8 hours, and 1.5 ± 0.16% at 48 hours post-infection (Figure 2). Our previous published results have shown that the lower percentage of SV40 minichromosomes containing RNAP II at 8 hours post infection is due the transcriptional switch between down regulation of early transcription and up regulation of late transcription occurring at approximately the 8 hour time point [15]. The percentage at 48 hours post-infection was similar but somewhat smaller than our previous report of approximately 10% at 48 hours post-infection [12]. The observed decrease was possibly due to differences in the age of the cells used in this analysis compared to our previous report. It was however closer to the 4% which has been previously reported using very different techniques [16].

In order to determine whether there was an effect on the number of SV40 minichromosomes carrying RNAPII following HDACi treatment, we then determined the percentage of SV40 minichromosomes that contained bound RNAPII after HDACi treatment. In these analyses we first determined whether there was any effect on the size of the pool of minichromosomes resulting from the HDACi treatment. While we noted that there was no significant effect on the overall size of the pool of SV40 minichromosomes following HDACi treatment at 8 hours and 48 hours there was a small effect at 30 minutes compared to the corresponding untreated control. At 30 minutes post-infection we observed C_T _values of approximately 16.9 in untreated samples and a C_T _value of approximately 15. 8 in the HDACi treated samples, at 8 hours post-infection C_T _values of approximately 15.3, and at 48 hours post-infection C_T _values of approximately 15.5. While treatment with the HDACi inhibitors appeared to slightly increase the uptake of virus at 30 minutes post-infection, the similarity in the C_T _values of the total pool of minichromosomes from the treated and untreated SV40 infected cells at the other times indicated that HDACi treatment was not significantly affecting the overall size of the pool of minichromosomes present at those times.

As shown in Figure 2 we observed approximately a tenfold increase in the percentage of SV40 minichromosomes carrying RNAPII following HDACi treatment at each time in the infection. In the pool of SV40 minichromosomes isolated 30 minutes post-infection the percentage of minichromosomes carrying RNAPII increased from 0.84 ± 0.19% in the untreated control to 10.23 ± 1.99% following NaBu treatment and 12.42 ± 2.17% following TSA treatment. Similarly, at 8 hours post-infection the values increased from 0.04 ± 0.01% in the control to 0.72 ± 0.21% following NaBu treatment and 1.27 ± 0.14% following TSA treatment. Finally, at 48 hours post-infection the percentages of SV40 minichromosomes carrying RNAPII increased from 1.5 ± 0.16% in the control to 9.47 ± 1.29% following NaBu treatment and 13.63 ± 1.5% following TSA treatment.

Since the size of the pool of SV40 minichromosomes did not change appreciably following HDACi treatment, the increase in SV40 minichromosomes carrying RNAPII appeared to result from a corresponding reduction in SV40 minichromosomes undergoing some other biological processes. This increase in the percentage of SV40 minichromosomes capable of transcription suggested that HDAC activity might play a critical role in controlling the biological fate of SV40 minichromosomes.

### 2.3. HDACi treatment inhibits SV40 replication

**Figure 3:**
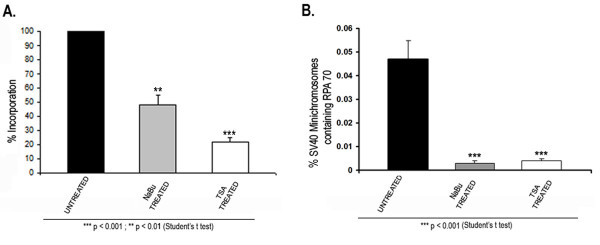
**Effects of HDACi treatment on SV40 Replication**. **(a) **BSC-1 cells infected for 48 hours with wild type 776 SV40 virus were either untreated or treated with 250 μM NaBu or 120 nM TSA and radiolabeled with *methyl*-[^3^H] thymidine. SV40 DNA was isolated and *methyl*-[^3^H] thymidine incorporation into newly replicating DNA was counted in a liquid scintillation analyzer and is represented as the percentage of *methyl*-[^3^H] thymidine incorporation relative to untreated cells. Data represented in the graph is Mean ± S.E. (n = 6). **(b) **48 hour SV40 minichromosomes either untreated or treated with 250 μM NaBu or 120 nM TSA were immune selected using 10 μl of antibody against RPA70. Data is expressed as percentage of SV40 minichromosomes containing RPA70 and represent the average of three independent experiments.

Since SV40 replication is an important biological process occurring at 48 hours post-infection, we hypothesized that the increase in SV40 minichromosomes carrying RNAPII at this time might cause a corresponding decrease in the SV40 minichromosomes capable of replication. In order to test this hypothesis, we first measured the incorporation of tritiated thymidine into total SV40 DNA in HDACi treated or untreated infected cells. As shown in Figure 3A, we observed a significant reduction in the amount of tritiated thymidine incorporated into the SV40 DNA from treated cells compared to untreated controls. For example, incorporation of tritiated thymidine was reduced to 48 ± 7% following treatment with NaBu and to 22 ± 3% following treatment with TSA. The reduction in the incorporation of the radiolabeled thymidine into SV40 DNA following HDACi treatment indicated that there was significantly less replication occurring following treatment with the inhibitors.

In order to confirm that replication was affected by HDACi treatment, we determined whether the percentage of SV40 minichromosomes carrying RPA70, a protein absolutely required for replication [17], was reduced following HDACi treatment. SV40 minichromosomes were harvested from HDACi treated or untreated infected cells 48 hours post-infection and subjected to a ChIP analysis using antibodies to RPA70 followed by real time PCR quantitation. As shown in Figure 3B, approximately 0.047 ± 0.008% of the minichromosomes isolated from untreated SV40 infected cells at 48 hours post-infection contained bound RPA70 and were presumably undergoing replication. Following treatment with NaBu and TSA the percentage of minichromosomes which contained RPA70 decreased to 0.003 ± 0.001% and 0.004 ± 0.001% respectively.

The inhibition in the uptake of tritiated thymidine and reduction in the percentage of SV40 minichromosomes containing bound RPA70 indicated that HDAC activity played a role in determining the biological fate of the SV40 minichromosomes as suggested by the results with transcribing SV40 minichromosomes described above.

### 2.4. Inhibition of histone deacetylation results in an increase in occupancy of RNAPII on SV40 minichromosomes

While the increase in SV40 minichromosomes which carry RNAPII described above could account for the observed stimulation of early and late transcription, it is also possible that the stimulation of transcription occurred at least in part as a result of the presence of more RNAPII transcription complexes on each of the transcribing SV40 minichromosomes. In order to test this hypothesis, we determined the occupancy of RNAPII on SV40 minichromosomes isolated at 30 minutes, 8 hours, and 48 hours post-infection from HDACi treated SV40 infected cells using an ISF analysis and compared the results to the occupancy in untreated SV40 minichromosomes [15]. In an ISF analysis SV40 minichromosomes containing a protein of interest such as RNAPII are bound to agarose in a typical ChIP procedure by an antibody which recognizes the protein. The minichromosomes which are bound to agarose are then fragmented by sonication. The DNA present in fragments which remain bound to the agarose and the DNA in fragments which are solubilized by the sonication are then amplified by PCR with primers that recognize sites of interest in the SV40 genome. The occupancy of a protein in a site of interest is defined as the percentage of the total DNA amplified from the site which remained bound to the agarose after the sonication.

The results of this analysis are shown in Table 1. In our initial experiments we compared treated and untreated minichromosomes isolated in parallel in three separate preparations. The untreated controls showed the same amount of RNAPII occupancy as our previous published results [15]. In order to obtain similar statistical significance for the treated samples we prepared additional samples in parallel which had been treated with either NaBu or TSA (n = 5).

**Table 1:** Relative occupancy of RNA Polymerase II on the SV40 genome after treatment with HDACi.

		**30 Min**	**8 Hour**	**48 Hour**
**EARLY**	*Untreated*	63 ± 1.5	43 ± 1.5	42 ± 2.0
	*NaBu Treated*	72 ± 2.0	67 ± 1.0*	54 ± 2.5
	*TSA Treated*	78 ± 1.5	65 ± 2.0*	57 ± 1.0

**LATE**	*Untreated*	34 ± 2.0	51 ± 3.0	52 ± 2.5
	*NaBu Treated*	40 ± 1.0	58 ± 2.0	84 ± 1.0**
	*TSA Treated*	39 ± 1.5	64 ± 1.0	89 ± 2.5**

**PROMOTER**	*Untreated*	51 ± 1.5	28 ± 3.5	60 ± 0.5
	*NaBu Treated*	53 ± 2.0	51 ± 1.0*	62 ± 3.0
	*TSA Treated*	57 ± 1.0	59 ± 2.5*	67 ± 1.5

**P < 0.001; * P < 0.05 (Student's t test). Untreated SV40 minichromosomes or SV40 minichromosomes treated with 250 μM NaBu or 120 nM TSA were isolated from cells infected with 776 wild-type virus for 48 hours. Purified SV40 minichromosomes were immunoprecipitated with 10 μl antibody to RNA polymerase II using the ISF technique and PCR amplified using primer sets to the early, late and promoter regions. Data in the table is represented as Mean ± S.E (n = 5 for treated samples and n = 3 for untreated control samples).

At 30 minutes post-infection HDACi treatment had relatively little effect on RNAPII occupancy in SV40 minichromosomes compared to untreated controls. Occupancy in the early region was 72 ± 2.0% (NaBu) and 78 ± 1.5% (TSA) compared to 63 ± 1.5%. Occupancy in the late region was 40 ± 1.0% (NaBu) and 39 ± 1.5% (TSA) compared to 34 ± 2.0%. Occupancy in the promoter was 53 ± 2.0% (NaBu) and 57 ± 1.0% (TSA) compared to 51 ± 1.5%.

However, at 8 hours post-infection occupancy by RNAPII of the early region and promoter but not the late region were significantly increased following HDACi treatment. Occupancy in the early region was 67 ± 1.0% (NaBu) and 65 ± 2.0% (TSA) compared to 43 ± 1.5%. Occupancy in the promoter was 51 ± 1.0% (NaBu) and 59 ± 2.5% (TSA) compared to 28 ± 3.5%. Occupancy in the late region was 58 ± 2.0% (NaBu) and 64 ± 1.0% (TSA) compared to 51 ± 3.0%.

At 48 hours post-infection HDACi treatment resulted in a substantial increase in RNAPII occupancy in the late region with less effect on the early region and no effect on the promoter. Occupancy in the late region was 84 ± 1.0% (NaBu) and 89 ± 2.5% (TSA) compared to 52 ± 2.5%. Occupancy in the early region was 54 ± 2.5% (NaBu) and 57 ± 1.0% (TSA) compared to 42 ± 2.0%, while occupancy in the promoter was 62 ± 3.0% (NaBu) and 67 ± 1.5% (TSA) compared to 60 ± 0.5%.

The large increase in RNAPII occupancy on the late region at 48 hours post-infection in conjunction with the continued high occupancies in the other regions of the genome indicated that there was an overall increase in the number of RNAPII transcription complexes which could contribute to the increase in late mRNA observed at this time. The large increase in RNAPII occupancy in the promoter at 8 hours post-infection suggested that histone deacetylation played a role in the down-regulation of early transcription which normally occurred at this time.

### 2.5. HDAC inhibition does not affect binding of p300 to RNAPII in transcribing minichromosomes

Since p300 was associated with RNAPII and hyperacetylated H4 and H3 in the coding regions of SV40 minichromosomes during transcription [12] and was absolutely necessary for SV40 transcription [12], we wondered whether HDACi treatment which typically results in increased histone hyperacetylation would allow the RNAPII transcription complexes in a coding region to function without associated p300. In order to address this question, SV40 minichromosomes were isolated from HDACi treated or untreated infected cells and the minichromosomes subjected to an Immune Selection Fragmentation followed by immunoprecipitation (ISFIP)/Re Chromatin Immunoprecipitation (ReChIP) analysis [12,15]. In an ISFIP/ReChIP analysis SV40 minichromosomes containing a protein of interest are immune selected with antibody to the protein and the minichromosomes fragmented by sonication as in an ISF analysis. The chromatin fragments which were originally bound to agarose are then eluted, and the eluted fragments and the solubilized fragments are each subjected to a second ChIP analysis with antibody to a second protein of interest. If the two proteins of interest are associated in the minichromosomes at some site in the genome, we would expect to find a PCR amplification product from the ReChIP portion of the analysis. In contrast if the two proteins are not associated we would expect to find an amplification product from the ISFIP portion of the analysis.

**Figure 4:**
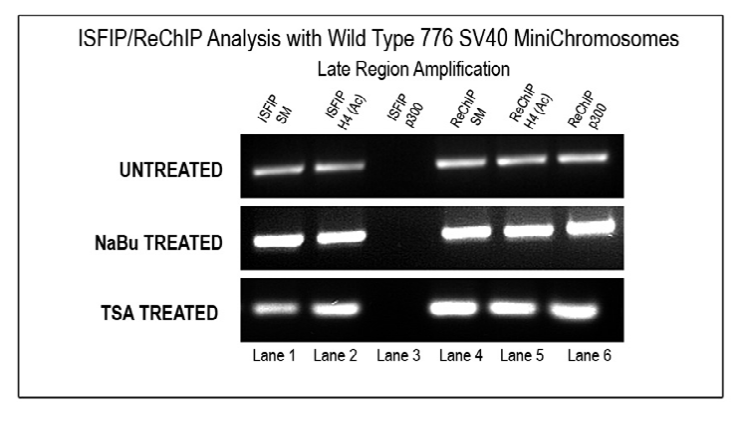
**Association of p300 with transcribing SV40 minichromosomes after HDACi treatment**. Unfixed SV40 minichromosomes treated with 250 μM NaBu or 120 nM TSA were isolated from cells infected with 776 wild-type virus for 48 hours, immunoprecipitated with RNAPII, and then subjected to an ISFIP/ReChIP analysis with antibody against p300. The samples were amplified by simplex PCR with primer sets to the late region. The position of the amplification product from the wild-type 776 DNA is indicated. Lane 1, ISFIP input fraction; lane 2, ChIP with 7.5 μl of hyperacetylated histone H4 antibody (ISFIP); lane 3, ChIP with 10 μl of p300 antibody (ISFIP); lane 4, ReChIP input fraction; lane 5, ChIP with 7.5 μl of hyperacetylated histone H4 antibody.

A typical example of this type of analysis is shown in Figure 4. Consistent with our previous publication using untreated minichromosomes [12], we observed p300 specifically associated with the RNAPII in the ReChIP fraction (lane 6) and not in the ISFIP fraction (lane 3). Hyperacetylated H4 which was used as a positive control was found in both the ReChIP and ISFIP fractions (lanes 5 and 2 respectively) as we have previously reported [12]. When minichromosomes from cells treated with NaBu or TSA were analyzed, similar results were obtained. p300 was again found associated with the ReChIP fraction (lane 6) and not the ISFIP fraction (lane 3), while hyperacetylated H4 was found associated with both fractions (lanes 5 and 2). These results indicated that p300 was still associated with RNAPII despite the fact that the histones present in the coding region had been extensively hyperacetylated by the HDACi treatment.

## 3. Discussion

Our current studies have shown that HDACi treatment can potentially stimulate SV40 transcription at the appropriate times during infection by increasing the number of minichromosomes carrying RNAPII through increased recruitment and by increasing the number of RNAPII molecules on the transcribed region of a minichromosome presumably through increased re-initiation. Interestingly, stimulation did not occur at all (the late genes at early times) or occurred only minimally (the early genes at late times) during the infection when the genes were naturally repressed, indicating that inhibition of HDAC activity was not sufficient by itself to overcome the normal repression of the genes at these times. The differential effects of HDACi treatment on the early and late genes at different time in infection were consistent with the observation that at any given time only between 2 and 20% of eukaryotic genes undergo significant stimulation of transcription following HDACi treatment [18].

HDACi treatment had no apparent effect on the association between p300 and RNAPII, which we have previously demonstrated to be necessary for transcription and histone hyperacetylation in the coding region of genes [12] suggesting that the targeting of HATs and HDACs to the RNAPII transcription complex occur independently.

The increased occupancy of the SV40 promoter and early coding region at 8 hours post-infection along with a two fold increase in early transcription after HDACi treatment suggested that HDACs may play a role in the down-regulation of SV40 early transcription which normally occurred at that time. Similar increases in occupancy were previously observed in the SV40 deletion mutant cs1085 which does not undergo down-regulation due to the inability of T-antigen to bind to its regulatory site in the promoter [15]. This suggested role for HDACs in SV40 regulation is consistent with the recent observation that T-antigen represses CBP-mediated transcription through interactions with HDAC1 [19].

Although there have been no previous studies of the effects of HDACi treatment on SV40 early and late expression during a lytic infection, the effects of NaBu on T-antigen expression in a non-permissive host [20] and SV40 transformed cells [21] have been investigated. In the former case NaBu appeared to cause a maximum stimulation of early transcription at later times in infection. In SV40 transformed cells NaBu appeared to cause approximately a five fold increase in T-antigen protein and mRNA, an increase similar to what was observed in our experiments.

Since HDACs are thought to be recruited to the promoters of many genes during repression of transcription [22], one way that HDAC inhibitors are thought to function is by blocking the recruitment of HDACs to promoters and thereby their repressive functions [23]. Our observation that HDAC inhibitors are capable of increasing the size of the pool of transcribing SV40 minichromosomes at early and late times is consistent with this suggested model. The fact that the HDAC inhibitors also cause a reduction in the size of the pool of replicating SV40 minichromosomes at late times suggests that HDAC activity may play a role in determining the biological fate of newly replicated SV40 minichromosomes. This suggestion that the fate of newly replicated SV40 minichromosomes may be determined in part by HDAC function is consistent with previous work which showed that treatment of SV40 infected cells late in infection with NaBu reduced the fraction of newly replicated minichromosomes which became committed to the encapsidation pathway [24].

Characteristically, many genes which are responsive to HDAC inhibitors contain specific response elements such as Sp1/Sp3 binding sites [22]. In this regard it is interesting to note that the SV40 regulatory region contains a series of Sp1 binding sites known as the 21 bp repeats which are required for transcription [25]. Moreover, we have shown previously that these Sp1 binding sites play a role in the nucleosomes phasing associated with the generation of a nucleosomes-free SV40 promoter region during initiation of transcription [26]. However, because of the complexity of the SV40 regulatory region and the presence of multiple transcription factor binding sites, we cannot exclude the possibility that interactions through other regulatory sequences may also be affected by HDACi treatment.

While the stimulation of transcription and increased occupancy of the late coding region at 48 hours post-infection following treatment correlates very well with a marked increase in hyperacetylated histones which we previously observed following NaBu treatment [12], it is also possible that the effects of treatment at 48 hours post-infection or other times is a result of an indirect effect of the HDAC inhibitors. As a consequence of their ability under certain conditions to deregulate specific genes including transcription factors such as Sp1 [27] or critical regulatory proteins such as waf1 [23], some of the effects of the HDAC inhibitors may be mediated through one or more of these aberrant regulatory factors acting on a gene of interest.

## 4. Materials and methods

### 4.1. Cells and viruses

SV40 virus and chromatin were prepared in the BSC-1 cell line of monkey kidney cells (ATCC). The 776 SV40 wild type virus was a gift from Dr Daniel Nathans.

### 4.2. Cell culture and infections

BSC-1 cells were maintained and infected at 10 pfu as previously described [12]. Treated cells were grown in the presence of 250 μM NaBu or 120 nM trichostatin A [Sigma] for 24 hour (in case of 8 hours and 48 hours post infection) or for 12 hours (in case of 30 mins post infection) prior to harvesting the minichromosomes.

### 4.3. Preparation of SV40 minichromosomes

SV40 minichromosomes from treated or untreated infected cells were harvested at 30 minutes, 8 hours or 48 hours post-infection and purified by glycerol gradient centrifugation as previously described [26,28]. Gradient fractions three, four and five, which contained SV40 minichromosomes, were combined for subsequent analysis.

### 4.4. Measurement of incorporation of Tritiated Thymidine into DNA

At 24 hours post-infection, SV40 infected BSC-1 cells were either treated with 250 μM of sodium butyrate (NaBu) or 120 nM of trichostatin A (TSA) or left untreated. Following a twenty four hour incubation, the HDACi treated or untreated cells were allowed to replicated in the presence of 500 μl of [*methyl*-^3^H-thymidine] (5 mCi, Amersham) for one hour. The cells were then washed twice in chilled PBS and extracted using the Hirt method of viral DNA extraction [29]. Purified SV40 DNA was dissolved in 20 μl of TE buffer. Five μl of the solution was placed in a scintillation vial with 3 ml of Ecoscint A scintillation solution (National Diagnostics) and counted in a liquid scintillation analyzer (Beckmann LS6500). The counts per minute (cpm) reflected the amount of radiolabel that was incorporated into the DNA.

### 4.5. Chromatin Immunoprecipitation and Immune Selection Fragmentation (ISF)

Untreated, NaBu treated and TSA treated SV40 minichromosomes were immunoprecipitated with 10 μl antibody to RNA Polymerase II (sc-900; Santa Cruz Biotechnology); 10 μl antibody to RPA 70 (sc-25376; Santa Cruz Biotechnology) or 7.5 μl antibody to hyperacetylated H4 (06–866 Upstate) using the reagents and protocol supplied by Upstate for the analysis of hyperacetylated H4 with minor modifications as previously described. In the final step of chromatin immunoprecipitation the pelleted agarose was resuspended in 200 μl of TE buffer. The resuspended agarose was sonicated and prepared for subsequent analysis as previously described [15,12,30].

### 4.6. Immune Selection Fragmentation followed by a second Immunoprecipitation (ISFIP)

Untreated, NaBu treated or TSA treated SV40 minichromosomes were immunoprecipitated with antibody to RNAP II as described above for the ISF procedure. In the final step of ISF, the soluble fraction obtained after sonication was used as the secondary input sample (200 μl) and immunoprecipitated with antibody to p300, (sc-584, Santa Cruz Biotechnology). The immunoprecipitation was carried out as described previously [12,30].

### 4.7. Re Chromatin Immunoprecipitation (Re-ChIP)

ReChIP was performed according to the procedure described by IJpenberg et al (2004) with minor modifications [31]. Untreated, NaBu treated or TSA treated SV40 minichromosomes were immunoprecipitated with antibody to RNAPII as described above for the ISF procedure. In the final step of ISF, the bound fraction was eluted twice with 200 μl Immunopure Gentle Ag/Ab Elution Buffer (Pierce). The bound fraction was incubated for 15-minute with elution buffer at room temperature and the eluted chromatin recovered by centrifugation. The eluates were pooled as the secondary input sample (200 μl) and immunoprecipitated with antibody to p300 (sc-584, Santa Cruz Biotechnology). The immunoprecipitation was carried out as described previously [15,12,30].

### 4.8. Preparation of DNA for PCR

Samples were prepared for PCR by phenol/chloroform extraction followed by ethanol precipitation in the presence of paint pellet co-precipitant (Novagen) as previously described [15,12,30]. Approximately 100 μl of protein A agarose eluates was purified using phenol/chloroform. The aqueous phase (125 μl) was added to a PCR tube that contained 3 μl of pellet paint co-precipitant and 12.5 μl of 3 M sodium acetate, pH 5.2 (Novagen). The samples were mixed and 280 μl of 100% ethanol added to each. Following 10-min incubation at room temperature, the samples were centrifuged at 8000 × g for 5 min and the supernatant discarded. The samples were washed with 70% ethanol, vortexed, incubated for 5 min, and then centrifuged at 8000 × g for 5 min. The supernatant was again discarded and the samples were dried in a vacuum.

### 4.9. PCR amplifications

DNA was amplified from three different regions of the SV40 genome (the early coding region, the late coding region and the promoter) in a Perkin-Elmer Model 480 thermal cycler using Ampli Taq Gold DNA Polymerase (Applied Biosystems) with primer sets 5'GCTCCCATTCATCAGTTCCA3' and 5' CTGACTTTGGAGGCTTCTGG3' for the amplification of the early region (nt 4540–4949), 5' CAGTGCAAGTGCCAAAGATC3' and 5'GCAGTTACCCCAATAACCTC3' for amplification of the late region (nt 1566–1878) and 5'GCAAAGCTTTTTGCAAAAGCCTAGGCCT3'and 5'CGAACCTTAACGGAGGCCTGGCG3' for amplification of the promoter region (nt 5168-420). A master mix containing all the required constituents was prepared according to the instructions supplied with the DNA polymerase in advance and kept at -20°C until required. Immediately before use, the master mix was thawed and a volume corresponding to 30 μl for each sample to be amplified was removed to prepare a working mix. The working mix was then prepared by adding the DNA polymerase to the master mix in the ratio of 0.5 μl per 30 μl of master mix. Following thorough mixing, the 30 μl of working mix was added to each previously prepared PCR tube containing a sample of template DNA to be amplified. The tubes were gently vortexed to suspend the pelleted DNA present in the tubes. When suspension was complete, the samples were overlaid with two drops of molecular biology grade mineral oil (Sigma). All previous manipulations were performed in a Nuaire biological safety cabinet Model NU_425-400. The samples were centrifuged for 1 min at 10,000 × g in an Eppendorf micro centrifuge, and the PCR amplifications were hot started by heating the tubes for 2 min and 30 sec at 95°C. The DNA was amplified for 45 cycles with each cycle consisting of annealing at 60°C for early region, 64°C for late region and 70°C for the promoter region for 1 min, DNA synthesis for 1 min at 72°C and denaturation at 95°C for 1 min.

### 4.10. Real Time PCR

DNA was amplified from the late region of the SV40 genome in a Cepheid Smart Cycler 2.0 System using the QuantiTect SYBR Green Real Time PCR Kit (QIAGEN, Valencia, CA) using the same primer sets described above. The DNA was amplified for 40 cycles with each cycle consisting of denaturation at 95°C for 30 sec, annealing at 64°C 30 sec, DNA synthesis for 1 min at 72°C.

### 4.11. Real Time Reverse Transcription PCR

Real Time RT-PCR analysis was performed using the QuantiTect SYBR Green RT-PCR kit (QIAGEN, Valencia, CA). Primer sets for the early and late coding region were same as described above. A master mix containing 25 μl of 2× QuantiTect SYBR Green RT-PCR Master Mix (1×), primer sets (0.5 μM of each primer), 0.5 μl of QuantiTect RT Mix (0.5 μl/reaction), template RNA (500 ng/reaction) and RNase free water to make the final volume of the mix to 50 μl. The reverse transcription step to synthesize the first strand cDNA was done at 50°C for 30 min, followed by a 15 min initial PCR activation step at 95°C to activate the HotStarTaq DNA Polymerase. The DNA was amplified for 40 cycles with each cycle consisting of denaturation at 94°C for 1 min, annealing at 60°C for early region GAPDH, 64°C for late region for 1 min, DNA synthesis for 1 min at 72°C and a final extension at 72°C for 10 min

### 4.12. Analysis of PCR amplification products

Following PCR amplification of the DNA samples, the products were separated on 2.4% submerged agarose gels (Sigma) by electrophoresis. The separated products were visualized by staining with ethidium bromide and electronically photographed using UVP GDS8000 Gel Documentation System (Ultra Violet Products).

### 4.13. Scanning densitometry

Quantitation of agarose gels was done with Molecular Analyst (Version 1.4) from Bio-Rad. Using Molecular analyst images were obtained by importing those that were captured with UVP GDS8000 Gel Documentation System. On importing the image, quantitation was performed with the Volume Analysis function to determine the percent volume of DNA bands of interest. The Local Background subtraction function was utilized to normalize background noise.

### 4.14. Data representation

Delta CT values were calculated as follows; ΔCT = CT (input material) - CT (ChIP sample with antibody to RNAP II [transcribing minichromosomes] or RPA 70 [replicating Minichromosomes]). ΔCT was expressed either as a percentage of minichromosomes containing the epitope for a particular antibody. Statistical analysis was performed using two tailed Student's t test.

### 4.15. Western blotting

Protein extracted from untreated, NaBu treated and TSA treated 48 hour wild type 776 SV40 virus infected cells were harvested and lysed using 1× RIPA buffer. Protein levels were determined with the Micro BCA™ Protein Assay kit (Pierce Biotechnology, Inc., Rockford, IL). The proteins were separated on a 4–20% PAGEr^® ^polyacrylamide gel (Cambrex BioScience Inc., Walkersville, MD) under denaturing conditions and electroblotted onto PVDF membrane (Millipore, Billerica, MA). Antibodies used for western blotting were goat polyclonal anti GAPDH (sc-20357); Santa Cruz Biotechnology; rabbit GST-fusion anti VP1 [32] and anti T Antigen.

## 5. Abbreviations

RNAP II: RNA Polymerase II, NaBu: sodium butyrate; TSA: trichostatin A; HDACi: HDAC inhibitors; ISF: immune selection and fragmentation; ISFIP: ISF followed by a second immunoprecipitation; ReChIP: re-chromatin immunoprecipitation; GAPDH: glyceraldehydes-3-phosphate dehydrogenase; VP1: viral protein 1; P.I.: post-infection

## 6. Competing interests

The author(s) declare that they have no competing interests.

## 7. Authors' contributions

LB performed the experiments and wrote the manuscript. BM edited the manuscript and provided input into the experiments. Both authors read and approved the final manuscript.
